# VIPS: Learning-View-Invariant Feature for Person Search

**DOI:** 10.3390/s25175362

**Published:** 2025-08-29

**Authors:** Hexu Wang, Wenlong Luo, Wei Wu, Fei Xie, Jindong Liu, Jing Li, Shizhou Zhang

**Affiliations:** 1 Xi’an Key Laboratory of Human–Machine Integration and Control Technology for Intelligent Rehabilitation, Xijing University, Xi’an 710123, China; wanghexu@xijing.edu.cn (H.W.); wuwei@xijing.edu.cn (W.W.); lijing@xijing.edu.cn (J.L.); 2 School of Information Science and Technology, Northwest University, Xi’an 710100, China; 202310355@stumail.nwu.edu.cn; 3 School of Computer Science, Northwestern Polytechnical University, Xi’an 710072, China; luowenlong@mail.nwpu.edu.cn; 4 Academy of Advanced Interdisciplinary Research, Xidian University, Xi’an 710071, China

**Keywords:** person search, UAV object tracking, cross-platform

## Abstract

Unmanned aerial vehicles (UAVs) have become indispensable tools for surveillance, enabled by their ability to capture multi-perspective imagery in dynamic environments. Among critical UAV-based tasks, cross-platform person search—detecting and identifying individuals across distributed camera networks—presents unique challenges. Severe viewpoint variations, occlusions, and cluttered backgrounds in UAV-captured data degrade the performance of conventional discriminative models, which struggle to maintain robustness under such geometric and semantic disparities. To address this, we propose **v**iew-**i**nvariant **p**erson **s**earch (VIPS), a novel two-stage framework combining Faster R-CNN with a view-invariant re-Identification (VIReID) module. Unlike conventional discriminative models, VIPS leverages the semantic flexibility of large vision–language models (VLMs) and adopts a two-stage training strategy to decouple and align text-based ID descriptors and visual features, enabling robust cross-view matching through shared semantic embeddings. To mitigate noise from occlusions and cluttered UAV-captured backgrounds, we introduce a learnable mask generator for feature purification. Furthermore, drawing from vision–language models, we design view prompts to explicitly encode perspective shifts into feature representations, enhancing adaptability to UAV-induced viewpoint changes. Extensive experiments on benchmark datasets demonstrate state-of-the-art performance, with ablation studies validating the efficacy of each component. Beyond technical advancements, this work highlights the potential of VLM-derived semantic alignment for UAV applications, offering insights for future research in real-time UAV-based surveillance systems.

## 1. Introduction

Unmanned aerial vehicles (UAVs), or drones, have undergone transformative advancements in autonomy, sensing, and AI integration, enabling their deployment across diverse sectors from precision agriculture and disaster response to smart city surveillance. The global drone market is expected to continue its growth trajectory, driven by demand for scalable, real-time data acquisition and analysis. A critical enabler of this growth is the fusion of artificial intelligence (AI) with UAV platforms, particularly in cross-platform perception systems where drones collaborate with ground-based sensors (e.g., CCTV, IoT devices) to achieve comprehensive environmental understanding.

**Figure 1:**
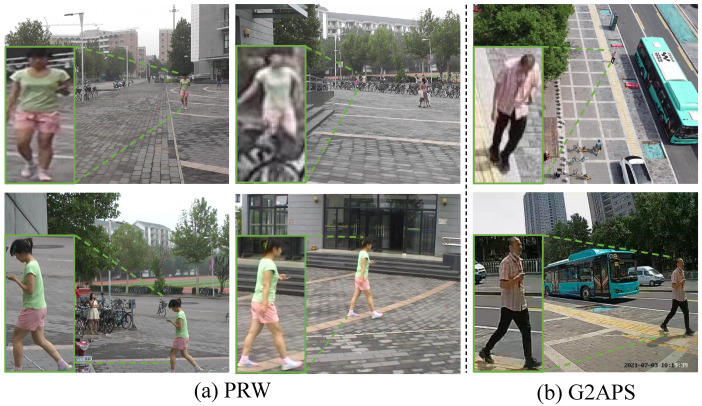
Person captured by different cameras in the PRW [1] and G2APS [2] datasets.The images in PRW are all from ground cameras, while the images in G2APS are from a ground camera and a UAV. A more detailed description of dataset characteristics is available in Section 5.

Such systems are increasingly vital for large-scale security monitoring, exemplified by applications like search-and-rescue operations, crowd behavior analysis, and cross-border surveillance. However, a fundamental challenge in these scenarios is cross-platform person search, seamlessly detecting and re-identifying individuals across heterogeneous camera networks comprising UAV-mounted and fixed ground cameras. As shown in Figure 1, a sharp contrast emerges between traditional same-platform datasets such as PRW, where camera views are relatively homogeneous and mostly ground-based, and cross-platform datasets like G2APS, which couple UAV-mounted aerial views with fixed ground cameras. The drastic viewpoint disparity in G2APS (e.g., top-down vs. frontal perspectives) results in pronounced geometric distortions, scale variations, and appearance shifts, thereby causing much higher false-negative rates compared to conventional ground-only benchmarks.

Existing person search methods mainly focus on the cooperation of the two subtasks, misalignment of scale, occlusion, and detector optimization; few works consider the view difference when matching persons across cameras. It may be because there is no camera annotation in one of the commonly used datasets, CUHK-SYSU [3]. However, its images are derived from hand-held cameras and movie snapshots and can still be viewed as coming from two different views. In addition, the PRW [1] dataset was captured by six different cameras, and the rich view information also needs to be further fully utilized. Recently, Zhang et al. [2] constructed the UAV- and camera-based person search dataset G2APS. The huge difference between the high altitude and ground view makes accurate person matching more difficult.

The visual–language pre-training model CLIP [4] unifies the two modes of image and text. It can learn semantic information from the prompt “A photo of a {xx} person” and match it with the corresponding image. It has the characteristics of zero-shot learning, so it is widely used in many downstream tasks such as image classification, object detection, and semantic segmentation. CLIP-ReID [5] applied CLIP to person ReID for the first time, learning the descriptor “A photo of a {xx} person” for each ID and guiding the image encoder to learn the semantic information in the image through the descriptor. Inspired by this, we use CLIP to learn the semantic information shared in images of different views and then embed the semantic information into image features to solve the problem of view difference.

In this work, we present view-invariant person search (VIPS), a novel two-stage framework that synergizes UAV-optimized detection with vision–language model (VLM) advancements to address cross-view person search challenges. The model consists of Faster R-CNN [6] and view-invariant person ReID (VIReID). VIReID uses CLIP-ReID as the baseline model. Like CLIP-ReID, VIReID has two training stages: one to learn ID descriptor text features and one for visual features. We observe that the presence of background and occlusion in the image introduces noise to the text feature, so we design a mask generator to eliminate the noise, allowing the model to learn more accurate text features. Furthermore, to reduce the difference between image features of the same ID under different views, we design the view prompt to embed different view information through a set of learnable embeddings. By inputting the view prompt and image patch embeddings into each encoder layer, the encoder learns view-invariant image features. To the best of our knowledge, VIPS provides pioneering contributions to AI-driven UAV applications. VIPS integrates CLIP’s semantic alignment for cross-platform person search, providing a dedicated solution for UAV-induced viewpoint discrepancies.

We conduct comprehensive evaluations on both person search benchmarks and traditional person re-identification benchmarks. The experimental results demonstrate that our proposed methods can significantly outperform existing state-of-the-art methods. The visualization of the feature mask further illustrates that our method can guide the model to focus on more discriminative regions. The contributions of this paper are summarized as follows:

- We propose a novel viewpoint-invariant person search (VIPS) framework leveraging CLIP’s semantic alignment for UAV and cross-camera scenarios;
- We propose a mask generator to suppress noise in UAV-captured images, enhancing text-guided feature learning and view prompts to encode camera perspectives into visual features, reducing viewpoint discrepancy;
- Extensive experiments on five benchmark datasets demonstrate the superiority of VIPS, establishing a new state-of-the-art method in UAV-based person search tasks.

The rest of this article is organized as follows: We review the related works in Section 2 and overview preliminary works in Section 3. The proposed framework and the optimization procedure are detailed in Section 4. Section 5 highlights the experimental evaluations. Section 6 draws the conclusion.

## 2. Related Work

In this section, we briefly review the related literature on person search and prompt learning, with a particular emphasis on the unique challenges posed by cross-view scenarios.

### 2.1. Person Search

Person search aims to locate and identify the target person from scene images by combining two subtasks: person detection and person re-identification (ReID). Existing methods are typically categorized into two-step methods and end-to-end frameworks. The two-step approach trains detection and ReID models independently, while the end-to-end approach integrates both tasks with a shared backbone.

Two-step methods often emphasize improving ReID performance through detection-aware mechanisms. For instance, TCTS [7] and IGPN [8] select query-like proposals to align detector outputs with ReID objectives. RDLR [9] further refines proposals by imposing ReID supervision to enhance identification. In contrast, end-to-end methods focus on resolving the conflict between detection and identification objectives. DMRN [10] structurally decouples the two tasks, while AlignPS [11] and HKD [2] employ strategies such as ReID-first optimization and head-level knowledge distillation to mitigate task interference and boost performance.

Despite these advances, most prior works [7,8] assume viewpoint consistency and primarily target ground-view scenarios. This assumption severely limits their generalizability to more complex settings, such as cross-view person search between UAV and ground platforms. In these cases, drastic viewpoint discrepancies, scale variations, and domain-specific noise (e.g., occlusion, motion blur, and background clutter) substantially degrade model robustness. A ReID feature extractor trained on one platform often fails to transfer across platforms, leading to misalignment in the embedding space and degraded search accuracy.

Several recent studies [2,12] have proposed benchmarks for cross-platform or cross-view person search. However, most methods still lack mechanisms to explicitly exploit platform-specific cues for learning view-invariant semantic representations. Our VIPS framework directly addresses this gap by introducing view-aware semantic prompts. By explicitly embedding platform-specific cues into the representation learning process, our approach captures discriminative yet view-robust semantics that generalize across cross-platform scenarios. This design effectively mitigates severe appearance variations, scale discrepancies, and background clutter inherent in such settings, thereby positioning our work as a targeted and scalable solution for person search under large cross-platform divergences.

### 2.2. Prompt Learning

Prompt learning has recently emerged as an effective paradigm for adapting large pre-trained models to downstream tasks. In natural language processing, carefully designed prompts are used to guide pre-trained models in specific tasks [13,14,15,16,17]. For instance, CLIP [4] connects images and natural language through the template “A photo of a {object}”. To reduce the reliance on manual prompt engineering, CoOp [18] introduces context optimization to automatically learn suitable prompt tokens. Later, Zhou et al. [19] designed conditional prompts that adapt to each input image, alleviating the bias toward seen classes. In the vision domain, VPT [20] uses learnable visual prompts to efficiently adapt pre-trained vision transformers to diverse tasks.

Inspired by VPT, we design learnable view prompts to represent platform-specific information. By incorporating view cues into image representations, our approach explicitly accounts for view discrepancies and learns features that remain robust under drastic cross-platform variations.

## 3. Preliminaries

### 3.1. Overview of CLIP-ReID

CLIP-ReID [5] applies CLIP [4] for the person ReID task. It consists of a text encoder and an image encoder, implemented by ViT-B/16 [21] *“A photo of a ${\left[X\right]}_{1}\;$${\left[X\right]}_{2}\;$${\left[X\right]}_{3}$…$\;{\left[X\right]}_{L}$ person*” for each person ID, where [X] represents a learnable text embedding.

CLIP-ReID adopts a two-stage training strategy. For the $k_{th}$ image with ID $y_{k}$, the model learns the text feature $T_{k}$ of the ID descriptor in the first stage. Then the model learns the visual feature $V_{k}$ under the supervision of $T_{k}$ and $y_{k}$.

In the first stage, two contrast losses are used:

(1) $$L_{i2t}\left(k\right)=-\log\frac{\exp(S\left(V_{k},T_{k}\right))}{\sum_{x=1}^{B}\exp\left(S\left(V_{k},T_{x}\right)\right)},$$

(2) $$L_{t2i}\left(y_{k}\right)=\frac{-1}{\left|Q(y_{k})\right|}\sum_{q\in Q(y_{k})}\log\frac{\exp(S\left(V_{q},T_{y_{k}}\right))}{\sum_{b=1}^{B}\exp\left(S\left(V_{b},T_{y_{k}}\right)\right)},$$

where S(·,·) represents the cosine similarity, *B* represents the batch size, $Q(y_{k})$ represents the index of images corresponding to $T_{y_{k}}$ in the batch, |·| represents the number of elements.

In the second stage, a cross-entropy loss is designed:

(3) $$L_{i2tce}\left(k\right)=-\sum_{j=1}^{N}q_{j}log\frac{exp(S\left(V_{k},T_{y_{j}}\right))}{\sum_{y_{b}=1}^{N}exp\left(S\left(V_{k},T_{y_{b}}\right)\right)},$$

where $q_{j}=\left(1-\epsilon \right)\delta_{j,y}+\frac{\epsilon }{N}$ represents the true probability distribution of class *j* and N represents the number of IDs.

CLIP-ReID successfully applies the visual language pre-training model to the person ReID task in a two-stage training method. The visual features extracted by CLIP contain rich semantic information, so we built a two-step personnel search framework, VIPS, with the help of CLIP-ReID. We use text features shared under different views to guide the model to learn view-invariant visual features and then align person features under different views.

### 3.2. Overview of Faster R-CNN

Faster R-CNN is an end-to-end framework composed of three key modules: a feature extractor $M_{f}$, a Region Proposal Network (RPN) $M_{rpn}$, and an RoI head $M_{roi}$. Given an image *I*, the feature extractor $M_{f}$ produces a dense convolutional feature map F. The RPN $M_{rpn}$ then slides a small network over F to generate, for of *k* predefined anchors at each location, an objectness score $p_{i}$ and a set of box offsets $l_{i}=\left(l_{x},l_{y},l_{w},l_{h}\right)$. After non-maximum suppression, the top proposals are reshaped by RoIAlign into fixed-size tensors and passed to the RoI head $M_{roi}$, which yields class probabilities $c_{i}\in R^{|C|+1}$ (|C| foreground classes plus one background) and refined box coordinates $l_{i}$.

Training relies on a multi-task loss. The RPN loss can be formulated as

(4) $$\begin{array}{cc} L_{RPN}\left(p,p^{*},l,l^{*}\right)\; & =\frac{1}{N_{cls}}\sum_{i}L_{cls}\left(p_{i},p_{i}^{*}\right)+\frac{1}{N_{reg}}\sum_{i}p_{i}^{*}\;L_{reg}\left(l_{i},l_{i}^{*}\right)\;, \end{array}$$

where $L_{cls}$ denotes cross-entropy classification loss, $L_{reg}$ is the smooth-$\ell_{1}$ regression loss, $p_{i}^{*}\in \left\{0,1\right\}$ and $p_{i}=1$ indicate the corresponding proposal region is positive (containing foreground objects), and $c_{i}^{*}$, $l_{i}^{*}$ are the ground-truth class labels and box regression targets, respectively. The RoI head loss can be formulated as

(5) $$\begin{array}{cc} L_{ROI}\left(c,c^{*},l,l^{*}\right)\; & =\frac{1}{N_{cls}}\sum_{i}L_{cls}\left(c_{i},c_{i}^{*}\right)+\frac{1}{N_{reg}}\sum_{i}p_{i}^{*}\;L_{reg}\left(l_{i},l_{i}^{*}\right)\;. \end{array}$$

The overall objective combines these two terms

(6) $$L=L_{RPN}+L_{ROI}\;.$$

Our system uses a trained Faster R-CNN to generate person bounding boxes, which are then cropped and resized for the ReID stage.

### 3.3. Vision Transformer

We adopt a Vision Transformer (ViT) as our visual feature encoder of VIPS. For an image with size H×W×3, ViT [21] divides it into $N_{p}$ image patches of P×P and encodes together with the position embedding into a d-dimensional vector $e_{i}\in R^{d}$. There are 12 encoder layers in ViT-B/16. We use $E_{i}=\left\{e_{i}^{j}\in R^{d}|1\leq j\leq N_{p}\right\}$ to represent the patch embeddings sent to the $i_{th}$ layer $L_{i}$. Then ViT is formulated as

(7) $$\begin{array}{cc} \left[x_{1},E_{1}\right] & =L_{1}\left(\left[CLS,E_{0}\right]\right), \end{array}$$

(8) $$\begin{array}{cc} {}[x_{i},E_{i}] & =L_{i}\left(\left[x_{i-1},E_{i-1}\right]\right)\;i=2,3,\ldots 12, \end{array}$$

where $x_{i}\in R^{d}$ represents the embedding of CLS, and [·,·] represents stacking two vectors.

## 4. View-Invariant Person Search

In this paper, we propose a two-step view-invariant person search model (VIPS). It consists of Faster R-CNN [6] and the view-invariant person ReID model VIReID. Faster R-CNN predicts the location of persons in scene images. VIReID aligns the image features of different views by learning the semantic information shared by the images of the same ID and learns the view-invariant features with the help of the view prompt, thereby solving the view problem.

### 4.1. View-Invariant Person ReID

Although CLIP-ReID has achieved satisfactory performance, it uses image features as guidance when learning text features in the first stage. If the image contains too much background or occlusions, there will be noise in the text features. In addition, CLIP-ReID can only rely on text features to align persons in different views, and the effect is limited by the performance of the text encoder.

**Figure 2:**
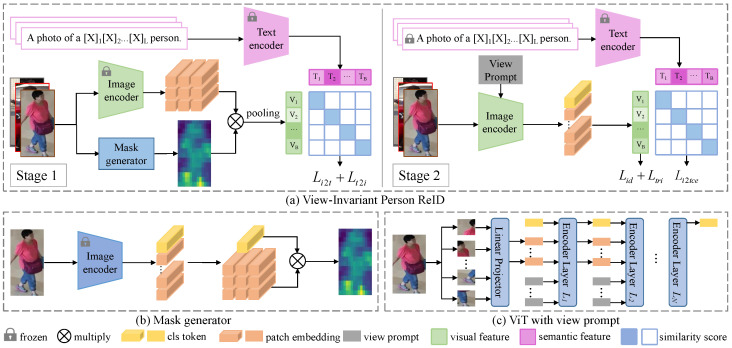
Overview of view-invariant person ReID (**a**). We use CLIP-ReID as the baseline model and propose a mask generator (**b**) and view prompt (**c**) to learn view-invariant features.

To overcome these challenges, we propose the view-invariant ReID (VIReID) module, which enhances CLIP-ReID with two novel UAV-specific components. Figure 2 shows the overview of VIReID. To learn more robust text features, we design a mask generator to generate a human foreground mask, which eliminates noise in image features. To further improve the cross-view alignment ability, we propose the view prompt at the image encoder level to further reduce the differences in person features under different views of the same ID.

#### 4.1.1. Mask Generator

The first stage learns the text feature of the ID descriptor through the contrast loss between the image feature and the text feature. The background and occlusions in the image will bring noise to the text feature. Weighting the image feature with a human foreground mask can effectively eliminate background and occlusions. As shown in Figure 2b, we design a mask generator implemented by an image encoder.

The last encoder layer of the mask generator obtains feature $F^{G}=\left\{x_{12}^{G},E_{12}^{G}\right\}$, where the patch embedding $E_{12}^{G}\in R^{N_{p}\times d}$. In order to generate the mask, we rearrange $E_{12}^{G}$ as $\left\{e_{12,m,n}|m=1,\ldots ,\frac{H}{P},n=1,\ldots ,\frac{W}{P}\right\}$, and $E_{12}^{G}\in R^{\frac{H}{P}\times \frac{W}{P}\times d}$. Then we calculate the cosine similarity of each patch embedding and CLS to obtain the mask $M\in R^{\frac{H}{P}\times \frac{W}{P}}$:

(9) $$M_{m,n}=S\left(e_{12,m,n},x_{12}\right),$$

where S(·,·) represents the cosine similarity.

At the same time, the image encoder I extracts visual features $F^{I}=\left\{x_{12}^{I},E_{12}^{I}\right\}$ of the input image. After reshape $E_{12}^{I}$ to the same size as $E_{12}^{G}$, it is weighted with the human body mask *M* to obtain the noise-free visual feature $V^{\prime }$:

(10) $$V^{\prime }=GAP\left(M⊙E_{12}^{I}\right),$$

where GAP represents global average pooling, and ⊙ denotes element-wise multiplication.

When using $V^{\prime }$ as the supervision information of the text encoder in the first stage, the learned text features will only contain information about the human body. The model will provide more accurate semantic features for the second stage.

#### 4.1.2. View Prompt

In the second stage, the image encoder learns the semantic information shared in different views under the guidance of the noise-free text features, which helps to solve the view difference problem. However, this does not eliminate the interference of the view difference. Therefore, we need to learn view-invariant visual features to enhance the model’s robustness to changes in views.

Inspired by the task prompt in VPT [20], we design the view prompt to embed view information in images. It is fed into the encoder together with the embedding of image patches so that the learned CLS features contain view information and can show stronger robustness in the face of view differences.

In practice, we treat each camera as a kind of view. Assuming that there are $N_{c}$ different cameras in the dataset, we initialize the view prompt $P\in R^{h\times N_{c}\times N_{v}\times d}$, where $N_{v}$ represents the number of embeddings required for each view, h∈[1,12] represents the number of layers in which the view prompt is applied in the encoder.

As shown in Figure 2c, when we extract image features, we send the view prompt to the image encoder together with the CLS embedding and patch embeddings. It should be noted that the view prompt added in layers 1 to h of the encoder differs for each specific camera. Given an image from the $c_{th}$ camera, and letting $E_{0}$ denote the input patch embeddings of the image, the calculation process of image features is formulated as

(11) $$\begin{array}{cc} \left[x_{1},E_{1}\right] & =L_{1}\left(\left[CLS,E_{0},P_{1}\right]\right), \end{array}$$

(12) $$\begin{array}{cc} {}[x_{i},E_{i}] & =L_{i}\left(\left[x_{i-1},E_{i-1},P_{i,c}\right]\right)\;i=2,3,\ldots h, \end{array}$$

(13) $$\begin{array}{cc} {}[x_{i},E_{i}] & =L_{i}(\left[x_{i-1},E_{i-1}]\right)\;i=h+1,h+2,\ldots 12, \end{array}$$

where $P_{i,c}=\left\{p_{i,c,n}\in R^{d}|n\in N,1\leq n\leq N_{v}\right\}$ represents the view prompt set used to embed camera *c* in the $i_{th}$ encoding layer, and $L_{i}$ represents the *i*-th encoder layer.

With the help of the learnable view prompt, the image features learned by the image encoder will contain corresponding view information. This allows person features to show greater robustness when facing intra-class differences between persons with the same ID under different cameras.

### 4.2. Training

We train the model in a two-step approach. First, the person search dataset is changed into datasets in the form of object detection and person ReID based on annotation, and then Faster R-CNN and VIReID are trained on these two datasets, respectively.

The loss function of VIReID is consistent with CLIP-ReID. In the first stage, the image encoder, text encoder, and mask generator are frozen, and only the ID descriptor is updated. The loss function is

(14) $$L_{stage1}=L_{i2t}+L_{t2i}.$$

In the second stage, only the image encoder is trained using cross-entropy loss and triplet loss for optimization.

(15) $$L_{id}=-\sum_{j=1}^{N}q_{j}log\left(p_{j}\right),$$

(16) $$L_{tri}=max\left(d_{p}-d_{n}+\alpha ,0\right),$$

where $p_{j}$ is the probability distribution predicted by the model. $d_{p}$ and $d_{n}$ represent the cosine distance between the features of the positive pair and the negative pair, respectively, and α is a preset margin.

Finally, the total loss function of the second stage is

(17) $$L_{stage2}=L_{id}+L_{tri}+L_{i2tce}.$$

### 4.3. Relation to Prior Work

CLIP-ReID applies CLIP to the person ReID task and learns the text descriptor features and visual features of images. However, it is not suitable for directly solving the view difference problem in person search. Therefore, we introduce a mask generator and view prompt to CLIP-ReID, ultimately solving the view problem through shared semantic information between images and view-invariant visual features.

## 5. Experiments

In this section, we will introduce the datasets and metrics used in the experiments, as well as details in training. Finally, our experimental results are analyzed.

**Datasets** To comprehensively evaluate our approach, we conducted experiments on three benchmark person search datasets and three person ReID datasets, covering both conventional and UAV-specific scenarios, CUHK-SYSU [3], PRW [1], and G2APS [2]. CUHK-SYSU is a large-scale benchmark containing 18,184 images with 8432 identities, notable for its realistic search scenario, where targets must be identified from whole gallery images rather than pre-cropped boxes. PRW comprises 11,816 frames from 6 synchronized cameras with 932 identities, emphasizing real-world challenges in pedestrian retrieval, with comprehensive annotations for both bounding boxes and identities. G2APS is the first cross-platform person search dataset specifically designed for UAV–ground camera scenarios, containing 31,770 images of 2077 identities. Each identity appears in both ground and aerial views, making it particularly valuable for evaluating view-invariant methods. In addition, to further verify the effectiveness of VIReID, we also conducted experiments on person ReID datasets Market1501 [22], MSMT17 [23], and Occluded-Duke [24]. The detailed information for each dataset is summarized in Table 1.

**Evaluation Protocols** We adopt Mean Average Precision (mAP) and Cumulative Matching Characteristics (CMC) as the evaluation metrics. mAP measures the overall retrieval quality by averaging the precision over all query identities, while CMC evaluates the retrieval accuracy at different ranks. Specifically, Rank-*k* denotes the proportion of query images whose correct match appears within the top-*k* retrieved gallery results, i.e., $\mathrm{Rank}\text{-}k=\frac{\#\mathrm{queries}\;\mathrm{correctly}\;\mathrm{matched}\;\mathrm{within}\;\mathrm{top}\text{-}k}{\#\mathrm{total}\;\mathrm{queries}}$. mAP is calculated as the mean of average precision scores across all queries, where each average precision is obtained by integrating the corresponding precision–recall curve. Unless otherwise stated, we report mAP/CMC in percentage (%) and all “improvements” as absolute percentage points. For all datasets, we follow the standard train/test splits provided in their official protocols.

**Implementation Details** The model was built using Pytorch, and all experiments were conducted on the NVIDIA RTX 3090 GPU. The person ReID model uses CLIP-ReID [5] as the baseline model. All weights were initialized to the weights of CLIP-ReID pre-trained on Market-1501 [22]. The initial weights of the mask generator and image encoder were the same. The batch size was B=64, 120 epochs were trained in the first stage, and 240 epochs were trained in the second stage.

The number of learnable text embeddings in the ID descriptor *L* was set to 6, the image patch size *P* was 16, and the dimension *D* was 512. The size of the view prompt was set to $h=6,N_{v}=11$, and $N_{c}$ directly took the number of cameras in the dataset. It should be noted that CUHK-SYSU [3] does not have camera annotation, but the images in the camera and movie can be regarded as two different view styles, so for CUHK-SYSU, $N_{c}=2$.

### 5.1. Comparison with State-of-the-Art Methods

In this section, we compare the proposed VIPS with other person search SOTA methods in Table 2. In addition, we also compare VIReID with the SOTA method of person ReID, and the results are shown in Table 3.

**Person search** As can be seen from Table 2, the best methods among the end-to-end methods and the two-step methods are HKD [2] and TCTS [7]. We attribute this to the former alleviating the conflict between detection and identification subtasks through the head knowledge distillation strategy, while the latter makes the detection and recognition tasks more consistent by generating query-like proposals. However, VIPS significantly outperforms both of them. VIPS outperforms HKD by 2.8%, 2.9%, and 15.6% in mAP on the three datasets, respectively. Compared with TCTS, which is also a two-step method, VIPS obtains 9.5% and 4.3% mAP advantages on PRW and CUHK-SYSU, respectively. These results collectively demonstrate that viewpoint variation represents a fundamental challenge in person search that has been largely overlooked in previous works. Our approach successfully addresses this limitation through its novel integration of foreground-aware feature purification and view-conditioned adaptation, establishing a new paradigm for view-invariant person search that is particularly suited for aerial surveillance scenarios.

**Person ReID Performance** As demonstrated in Table 3, our proposed VIReID establishes new state-of-the-art performance across all three benchmark datasets. The method achieves remarkable mAP scores of 90.3%, 74.9%, and 60.2% on Market-1501, MSMT17, and Occluded-Duke, respectively, surpassing all existing CNN-based and ViT-based approaches. Notably, VIReID shows particularly strong performance on MSMT17 (74.9% mAP). We attribute these results to the inherent characteristics of MSMT17. MSMT17 is characterized by a complex multi-camera setup, wide-ranging viewpoints, and diverse environmental conditions, which closely mirror the cross-platform scenario. This comprehensive evaluation not only validates our technical innovations but also highlights the importance of viewpoint invariance as a critical research direction for UAV-based person recognition systems.

### 5.2. Ablation Study

In this section, we conduct thorough ablation experiments on PRW to explore the effectiveness of each proposed module.

**Effectiveness of mask generator and view prompt** We incrementally added the mask generator and view prompt modules to the baseline model to assess their individual and combined contributions. The experimental results are summarized in Table 4. The baseline model achieves an mAP of 55.5% and a top-1 accuracy of 81.0%. With the addition of the mask generator, the performance improves slightly to 55.8% mAP and 82.1% top-1 accuracy. Similarly, introducing the view prompt yields 56.0% mAP and 81.6% top-1. When both modules are integrated, the model reaches its best performance at 56.3% mAP and 82.6% top-1 accuracy, indicating gains of +0.8% mAP and +1.6% top-1 over the baseline. These results validate the complementary benefits of the two modules. The mask generator helps the text encoder focus on foreground semantics by filtering out background noise and occlusions, while the view prompt enhances the image encoder’s ability to learn robust visual representations across varying viewpoints.

**Analysis of Performance Heterogeneity Across Benchmarks** As shown in Table 2 and Table 3, the performance gains vary substantially across datasets. We attribute these results to the distinct characteristics of different datasets. Datasets with greater view diversity and geometric shifts (e.g., G2APS with UAV–ground pairs and MSMT17 with 15 cameras) exhibit the most significant improvements, as our proposed camera-conditioned prompts effectively bridge cross-view gaps. In contrast, relatively smaller gains are achieved on PRW and CUHK-SYSU, with fewer or more homogeneous cameras. Furthermore, the severe occlusion and clutter in G2APS and MSMT17 highlight the benefits of VIPS’s mask generator, which suppresses irrelevant regions. To further validate this, we conducted an ablation on MSMT17 (Table 5), showing that improvements remain stable across different time-of-day splits, while being slightly larger in complex indoor scenes, demonstrating VIPS’s effectiveness under challenging environments. The performance heterogeneity further confirms the strength of our proposed method under large cross-view and cluttered conditions.

**Figure 3:**
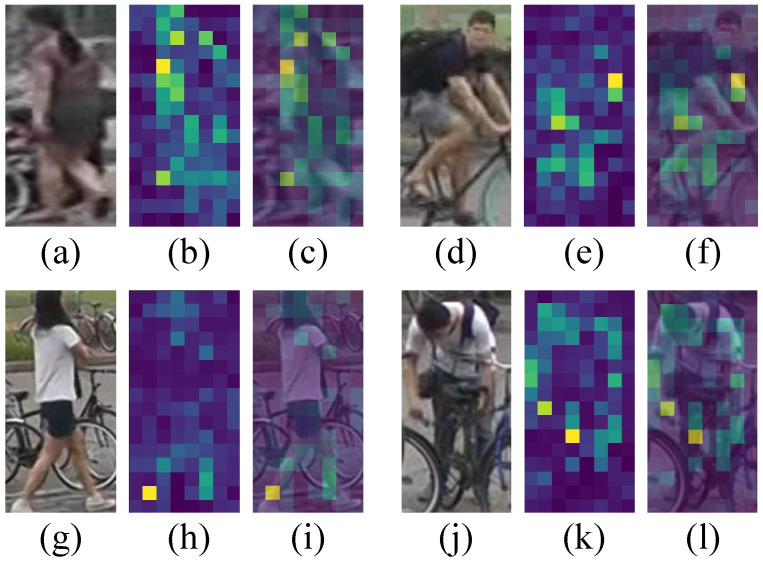
Mask of some person in PRW, where (**a**,**d**,**g**,**j**) represent the original image, (**b**,**e**,**h**,**k**) represent the mask. (**c**,**f**,**i**,**l**) represent the superposition of the mask and the original image.

**Mask visualization** Figure 3 presents visualizations of the generated foreground masks under different scenarios. In the first two examples, the scenes are relatively clean, with minimal background clutter and occlusion. In contrast, the latter two examples contain significant occlusions—primarily from the background and a bicycle, respectively. These visualizations demonstrate that the proposed foreground mask is capable of effectively suppressing irrelevant background regions and occluding objects, thereby enhancing the focus on salient targets. This is particularly valuable for UAV-based perception tasks, where dynamic environments and occlusions are common challenges.

**Figure 4:**
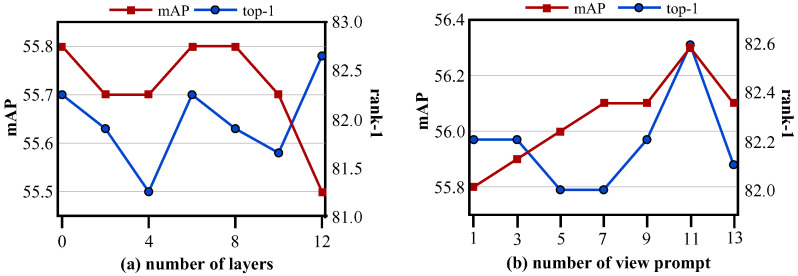
Effect of view-prompt depth *h* and token count $N_{v}$ on PRW performance. (**a**) Retrieval mAP and top-1 accuracy as *h* increases (with $N_{v}=1$). (**b**) Retrieval mAP and top-1 accuracy as ($N_{v}$) increases (with h=6).

**Hyper-parameter Sensitivity** As illustrated in Figure 4, we studied the impact of the two view-prompt hyper-parameters, prompt depth *h* and token count $N_{v}$. First, with $N_{v}$ fixed at 1, we varied *h* over {0,2,4,6,8,12} and observed that both mAP and top-1 accuracy peaked at h=6. Next, holding h=6 constant, we swept $N_{v}$ through {1,3,5,7,9,11,13}, finding the best retrieval performance at $N_{v}=11$. Consequently, we adopted h=6 and $N_{v}=11$ for all subsequent experiments.

**Figure 5:**
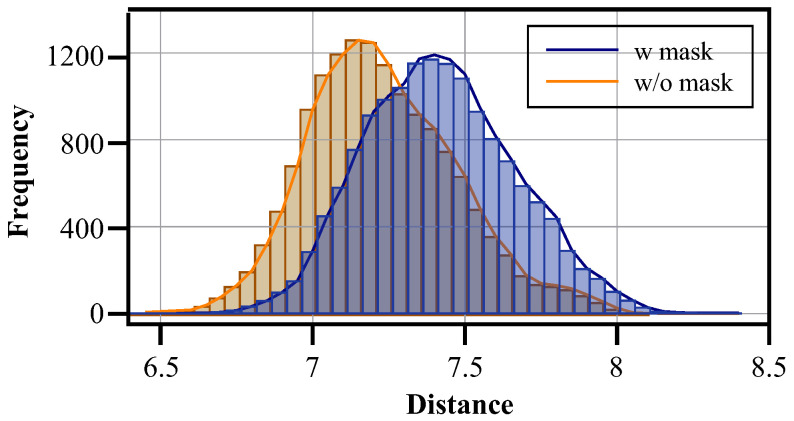
Euclidean distance distribution of image features and corresponding text features in the training set of PRW.

**Effectiveness of mask** Figure 5 shows the distance distribution between the visual features of the images in the training set and the corresponding text features. After applying the mask, the distance between textual features and visual features is significantly reduced. This shows that the mask can remove the noise in text features. In the second training stage, the image features contain more accurate semantic information, which helps the model solve the problem of view differences through the semantic information shared between different view images.

**Figure 6:**
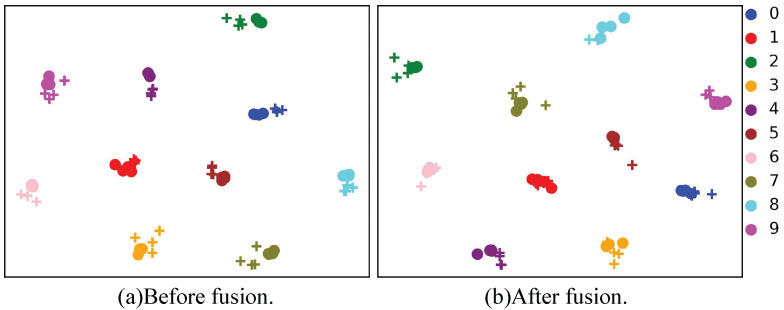
Distribution of image features in two views before (**a**) and after (**b**) fused with the view prompt. “o” and “+” represent the ground camera and UAV views, respectively, and the numbers represent the sample index rather than the ID.

**Visualization of view prompt effects** The G2APS dataset contains images in the ground camera and UAV views, with a very significant view difference. Therefore, we use t-SNE to visualize the image feature distribution under two different views before and after fusing the view prompt, and the results are shown in Figure 6. Before fusing the view prompt with the image feature, although the samples with the same ID are closely clustered, there is an obvious gap between the feature distributions of the two different views. After fusing, the image features of different views for each ID are brought closer. This demonstrates that the view prompt helps the model learn view-invariant visual features.

## 6. Conclusions

This paper addresses the critical yet understudied challenge of viewpoint differences in person search, a problem exacerbated in UAV-based surveillance where cross-camera perspective shifts severely degrade matching accuracy. We propose VIPS, a novel two-stage framework that combines Faster R-CNN with a view-invariant ReID module, leveraging CLIP’s vision-language paradigm to align features across viewpoints through shared semantic descriptors. To overcome noise from UAV-captured backgrounds and occlusions, we introduce a mask generator to purify text-guided feature learning. Furthermore, our view prompts explicitly encode camera perspectives into visual features, mitigating viewpoint divergence—an innovation particularly relevant for UAV multimodal applications where perspective robustness is essential. Extensive experiments demonstrate the effectiveness of our proposed method in resolving view differences. This work not only advances person search technology but also highlights the potential of vision–language models (VLMs) for UAV-centric tasks, advancing the application of new AI technologies in UAVs.

## Figures and Tables

**Table 1:** Statistics of the datasets used in our experiments. “#image” denotes the number of images, and “#ID” denotes the number of unique person identities. For person search datasets (CUHK-SYSU, PRW, and G2APS), the images are uncropped scene-level images containing multiple pedestrians, while for person ReID datasets (Market-1501, MSMT17, and Occluded-Duke), each image is a cropped pedestrian bounding box. In CUHK-SYSU, camera and movie images are treated as two distinct views. MSMT17 is a large-scale, multi-view dataset collected across 15 cameras under diverse indoor and outdoor conditions, featuring pronounced viewpoint differences that closely resemble cross-platform person search scenarios.

Dataset	Training Set		Test Set		#Cam
	#Image	#ID	#Image	#ID	
CUHK-SYSU	11,206	5532	6978	2900	2
PRW	5704	482	6112	450	6
G2APS	21,962	2077	9808	566	2
Market-1501	12,936	751	19,732	750	6
MSMT17	32,621	1041	93,820	3060	15
Occluded-Duke	15,618	702	19,871	1110	8

**Table 2:** Comparison of VIPS and all state-of-the-art person search methods on PRW, CUHK-SYSU, and G2APS datasets. The end-to-end methods are marked with *, and the best results are in bold. Param. (M) denotes the number of learnable parameters.

Method	Year	Param. (M)	PRW		CUHK-SYSU		G2APS	
			mAP	Top-1	mAP(M)	Top-1	mAP	Top-1
MGTS [25]	2018	-	32.6	72.1	83.0	83.7	-	-
CLSA [26]	2018	-	38.7	65.0	87.2	88.5	-	
RDLR [9]	2019	-	42.9	70.2	93.0	94.2	-	-
IGPN [8]	2020	-	47.2	87.0	90.3	91.4	-	-
TCTS [7]	2020	-	46.8	87.5	93.9	95.1	-	-
SeqNet * [27]	2021	48.4	46.7	83.4	93.8	94.6	34.0	44.5
AlignPS [11]	2021	42.2	45.9	81.9	93.1	93.4	27.0	34.7
OIM++ * [28]	2022	-	46.8	83.9	93.1	93.9	32.5	40.3
PSTR * [29]	2022	-	49.5	87.8	93.5	95.0	28.4	39.9
COAT * [30]	2022	37.0	52.5	86.0	93.7	94.1	40.3	50.5
HKD * [2]	2023	54.4	53.5	86.6	95.3	96.1	41.4	51.9
Faster+HOreid [31]	2023	95.6	55.6	**98.3**	97.4	98.0	52.6	62.2
SPNet-L * [32]	2024	-	54.2	89.0	95.8	96.3	-	-
UIL * [33]	2024	-	51.5	86.1	93.9	94.7	-	-
VIPS (ours)	-	168.2	**56.3**	82.6	**98.2**	**98.6**	**57.0**	**66.1**

**Table 3:** Comparison of VIReID and all state-of-the-art person ReID methods on three datasets. The best results are highlighted in bold.

Method	References	Market-1501		MSMT17		Occluded-Duke	
		mAP	Top-1	mAP	Top-1	mAP	Top-1
CNN as the backbone							
DRL-Net [34]	TMM 2022	86.9	94.7	55.3	78.4	50.8	65.0
LTReID [35]	TMM 2022	89.0	95.9	58.6	81.0	-	-
ETNDN [36]	TSCVT 2023	88.7	95.7	58.0	82.7	57.6	68.1
CLIP-ReID [5]	AAAI 2023	89.8	95.7	63.0	84.4	53.5	61.0
CGE [37]	PR 2023	90.1	95.6	65.9	85.1	-	-
ViT as the backbone							
DCAL [38]	CVPR 2022	87.5	94.7	64.0	83.1	-	-
PFD [39]	AAAI 2022	89.6	95.5	64.4	83.8	60.1	67.7
AAformer [40]	TNNLS 2023	87.7	95.4	63.2	83.6	58.2	67.0
CLIP-ReID [5]	AAAI 2023	89.6	95.5	73.4	88.7	59.5	67.1
RGANET [41]	TIFS 2023	89.8	95.5	72.3	88.1	-	-
PHA [42]	CVPR 2023	90.2	96.1	68.9	86.1	-	-
VIReID (ours)		**90.3**	**96.1**	**74.9**	**89.2**	**60.2**	**67.9**

**Table 4:** Performance comparison before and after adding the mask generator and view prompt to the baseline model. ✓ means adding it, and × means not adding it. The best results are highlighted in bold.

Mask Generator	View Prompt	PRW	
		mAP	Top-1
×	×	55.5	81.0
✓	×	55.8	82.1
×	✓	56.0	81.6
✓	✓	**56.3**	**82.6**

**Table 5:** Ablation study on MSMT17 under different query splits. The query set is divided by time of day into morning, noon, and afternoon subsets and by scene type into indoor and outdoor subsets, while the gallery set remains unchanged.

Methods	Time of Day						Scene			
	Morning		Noon		Afternoon		Indoor		Outdoor	
	mAP	Rank-1	mAP	Rank-1	mAP	Rank-1	mAP	Rank-1	mAP	Rank-1
Baseline	77.1	90.8	67.3	84.3	74.4	90.0	67.0	86.9	74.3	89.0
VIReID (ours)	78.5	91.1	69.1	85.1	76.0	90.4	69.8	87.9	75.6	89.4

## Data Availability

The original contributions presented in this study are included in the article. Further inquiries can be directed to the corresponding authors.
